# Hybrid black widow optimization with iterated greedy algorithm for gene selection problems

**DOI:** 10.1016/j.heliyon.2023.e20133

**Published:** 2023-09-14

**Authors:** Mohammed Alweshah, Yasmeen Aldabbas, Bilal Abu-Salih, Saleh Oqeil, Hazem S. Hasan, Saleh Alkhalaileh, Sofian Kassaymeh

**Affiliations:** a Prince Abdullah Bin Ghazi Faculty of Information and Communication Technology, Al-Balqa Applied University, Al-Salt, Jordan; b Department of Plant Production and Protection, Faculty of Agricultural Technology, Al-Balqa Applied University, Al-Salt, Jordan; c Department of Computer Science, King Abdullah II School of Information Technology, The University of Jordan, Amman, Jordan; d Software Engineering Department, Faculty of Information Technology, Aqaba University of Technology, Aqaba, Jordan

**Keywords:** Gene selection, Feature selection, Medical diagnosis, Iterated greedy algorithm, Black widow optimization, Metaheuristic hybridization

## Abstract

Gene Selection (GS) is a strategy method targeted at reducing redundancy, limited expressiveness, and low informativeness in gene expression datasets obtained by DNA Microarray technology. These datasets contain a plethora of diverse and high-dimensional samples and genes, with a significant discrepancy in the number of samples and genes present. The complexities of GS are especially noticeable in the context of microarray expression data analysis, owing to the inherent data imbalance. The main goal of this study is to offer a simplified and computationally effective approach to dealing with the conundrum of attribute selection in microarray gene expression data. We use the Black Widow Optimization algorithm (BWO) in the context of GS to achieve this, using two unique methodologies: the unaltered BWO variation and the hybridized BWO variant combined with the Iterated Greedy algorithm (BWO-IG). By improving the local search capabilities of BWO, this hybridization attempts to promote more efficient gene selection. A series of tests was carried out using nine benchmark datasets that were obtained from the gene expression data repository in the pursuit of empirical validation. The results of these tests conclusively show that the BWO-IG technique performs better than the traditional BWO algorithm. Notably, the hybridized BWO-IG technique excels in the efficiency of local searches, making it easier to identify relevant genes and producing findings with higher levels of reliability in terms of accuracy and the degree of gene pruning. Additionally, a comparison analysis is done against five modern wrapper Feature Selection (FS) methodologies, namely BIMFOHHO, BMFO, BHHO, BCS, and BBA, in order to put the suggested BWO-IG method's effectiveness into context. The comparison that follows highlights BWO-IG's obvious superiority in reducing the number of selected genes while also obtaining remarkably high classification accuracy. The key findings were an average classification accuracy of 94.426, average fitness values of 0.061, and an average number of selected genes of 2933.767.

## 1 Introduction

The development of successful diagnosis and classification relies heavily on gene expression data. Gene expression results are often repetitive and noisy, with just a small percentage of them presenting different profiles for various types of samples. As a result, in the field of bioinformatics, It's becoming more and more interesting to select highly discriminative genes from gene expression data [1]. A microarray is a microchip-based technique that is used to research gene sequences. This technology was created at the end of the 1990s and has since grown in popularity [2].

Gene expression data are a highly dimensional, large-scale type of data. Therefore, the gene selection (GS) process is applied to gene expression data as an important preprocessing stage for correct disease classification [3,4]. The major goal of GS is to keep the key features of large-scale databases while simplifying their quality. The selected genes are essential for identifying and classifying diseases [5,6]. However, several irrelevant genes are usually included in the generated gene expression, which makes disease detection more difficult. Also, according to experts in the field of AI, high dimensionality could deceive the learning algorithm and have a negative impact on the generalization mechanism because several noisy genes may be included in the learning mode [7,8]. Hence, FS is an important step in the classification process because it helps to minimize both the size and redundancy of gene expression data [9,10].

Feature selection involves choosing a subset of variables from the input that can effectively explain the input data [11,12]. The aim of FS is to eliminate noise or trivial effects and still produce good results for prediction. Feature selection helps users understand results, reduces computational time, mitigates the curse of dimensionality, and enhances predictor efficiency [13,14].

Feature selection has been recognized as a difficult problem for several years, and researchers have been trying to find the best way to improve the precision and productivity of FS [[15], [16], [17]]. It is now widely acknowledged that an AI strategy is needed to solve the FS problem. Scientists are now focusing on using another algorithm for the learning model to enhance the FS process. One type of algorithm that can be used to achieve this goal is a metaheuristic [18,19]. This type of algorithm can be employed as an optimizer for the learning model [20,21]. Metaheuristic algorithms have captured the interest of several researchers in recent years because these algorithms have the capacity to solve diverse problems in different fields [22,23]. Consequently, several recommendations have been made that metaheuristic algorithms in biology, math, or physics are influenced by almost distinct phenomena [24,25].

Numerous metaheuristic strategies have been suggested for the FS problem, such as the genetic algorithm (GA), GRASP, tabu search (TS), artificial bee colony (ABC), and harmony search (HS), among others [20,26]. Although several of these algorithms are used in this study, research is ongoing to discover new algorithms to solve the FS problem.

Among the techniques that have been used in this area are metaheuristic algorithms. Due to the increasing number of metaheuristics that have been proposed and their efficiency in achieving exact outcomes, metaheuristic algorithms are frequently used for optimization [27,28]. Nowadays, they are often used alone or paired with another method to ensure that the right solutions are found [29,30,31]. The analysis of microarray instruments and systems for pharmacogenomics testing and drug development is the subject of interest in this study. Therefore, in this study, a metaheuristic called the BWO will be applied to find more precise solutions for FS problems. The BWO algorithm, which was proposed by Hayyolalam and Kazem [32], has shown its strength when used in the field of optimization. It is now commonly used in complex optimization problems. BWO was applied for GS in two ways: it was employed in its original form, and it was hybridized with an iterated greedy (IG) algorithm, BWO-IG, to improve the BWO's local search capabilities.

In this paper, nine microarray GS datasets are used to test the effectiveness of the proposed approach. These benchmark datasets are from the GS data warehouse and have been used in a lot of the studies that are reviewed. These datasets are used to fully analyze and achieve further generalization of the findings. The nine datasets are MLL, Colon, Leukemia, leukemia_c3, Leukemia_c4, CNS, Ovarian, SRBCT, and lymphoma.

The key contributions of this study are as follows.

• Addressing the complexities and data imbalances inherent in gene selection in the context of microarrays
• presenting a simplified, computationally efficient approach to dealing with the conundrum of trait selection in microarray gene expression data.
• Use the Black Widow Optimization algorithm in the context of gene selection.
• Using two unique methodologies: variation of the Widow Optimization Algorithm and the hybrid BWO variant combined with the Iterated Greedy (BWO-IG) algorithm to process gene selection
• Improving the local search capabilities of the Widow optimization algorithm to promote more efficient gene selection.
• The proposed methods were applied to nine benchmark datasets obtained from the Gene Expression Data Repository, seeking empirical validation.

The rest of the paper includes: Section 2 begins with an examination of the works that are most relevant to this investigation. Sections 3, 4, 5 examine the BWO, the IG, and the recommended techniques for GS, respectively. In Section 6, the experiments and findings are presented and discussed. Finally, in Section 7, there is a conclusion.

## 2 Related work

### 2.1 A brief review on metaheuristic algorithms

Metaheuristic algorithms are a type of optimization approach that is frequently utilized in engineering, economics, science, and artificial intelligence to address complicated optimization issues. These approaches are intended to solve optimization problems with a wide search space and non-differentiable, noisy, or complex objective functions.

Metaheuristics work by directing the search process using a set of rules, heuristics, or probabilistic tactics, in contrast to classical optimization approaches that rely on gradient-based techniques for smooth and well-defined functions. They frequently give up the assurance that the global optimal will be discovered in favor of their prowess at navigating complex and varied problem environments.

In order to improve their exploration and exploitation techniques over time, metaheuristic algorithms can adapt and learn from the progress made throughout the search. They are ideal for optimization issues with potential for dynamic change or those without a straightforward mathematical description because of their versatility.

Because of their versatility, adaptability, and capacity to solve optimization issues satisfactorily in challenging and dynamic situations, metaheuristic approaches have been widely used to address a variety of optimization problems. The optimization problem's characteristics and the application's particular needs, however, will determine which algorithm is best.

In general, metaheuristic algorithms are classified as single-objective and multi-objective. In the **single-objective** area, there are several studies that are proposed in the literature. For instance, Kootanaee et al. [33] proposed a model that employs a hybrid strategy of an enhanced ID3 decision tree and a support vector machine. The evolutionary algorithm and multilayer perceptron neural networks are used to increase performance and accuracy. The planned effort aims to address financial statement fraud, which threatens the credibility of capital markets, corporate leaders, and even the audit profession. Financial statement fraud affects firms, governments, and investors. Nevertheless, there are many ways to recognize this issue, and auditors in particular struggle with their seeming inability to spot widespread fraud.

Two studies were put forward by Kassaymeh et al. [34,35] to address the problem of software defect estimation. In the first study, they used the Salp Swarm (SSA) algorithm as an alternative training technique for an artificial neural network (ANN). In the second, they hybridized the Simulated Annealing (SA) algorithm with SSA to enhance the optimization capabilities of SSA as a trainer for backpropagation neural networks. Metaheuristic algorithms have shown their strength in parameter optimizations as an alternate training approach to ANN, whose fundamental shortcoming is that its performance is entirely dependent on its initial weighting and bias. Dealing with software defect estimation (SDE) was the final objective.

Shirvani et al. [36], provides a hybrid meta-heuristic method to resolve scientific operations that are parallelizable on elastic cloud platforms because using a single strategy to address such complex issues would not produce the best results. A hybrid discrete particle swarm optimization (HDPSO) approach with three phases is presented to address this combinatorial problem. DPSO employed a hill climbing approach to avoid getting caught in ineffective traps and to strike a balance between exploration and exploitation.

The software effort prediction (SEP) and software test prediction (STP) issues were addressed by Kassaymeh et al. [37] by combining the salp swarm algorithm (SSA) and a backpropagation neural network (BPNN). These issues come up frequently in software engineering while attempting to identify the precise software resources required to complete a software project. The primary goal of the study was to employ the SSA trainer approach to increase prediction accuracy because the performance of BPNN completely depends on the initial parameter values.

In the **multi-objective** area, there are various studies that are proposed in the literature. For instance, Farzai et al. [38] create a novel solution to the difficult process of placing virtual machines (VMP) in cloud datacenters (DCs). While obtaining a high quality of service (QoS) is important for users, power consumption and resource waste are two objectives that providers should optimize. The problem that a user encounters in the cloud environment is network delay, which is impacted by shared bandwidth linkage between the apps of many users. They expand a hybrid multi-objective genetic-based optimization method to solve this combinatorial multi-objective issue.

Given that schedulers must take into account task interdependencies, underlying heterogeneity, cost variance, and different virtual machine (VM) configurations during the scheduling process, scheduling scientific processes on a hybrid cloud architecture is a difficult issue. So, with the intention of minimizing both financial cost and makepan, the problem of scientific workflow scheduling running on a hybrid cloud architecture is presented as a two-objective optimization problem. Shirvani et al. [39] proposed two-objective hybrid optimization based on simulation annealing and task iterative algorithms (BOSA-TDA) to address this discrete combinatorial problem, making use of two significant heterogeneous experimental approaches to completion time (HEFT) and coupling techniques to enhance the canonical SA.

On the other hand, there are **continuous optimization** and **discrete optimization** methods that have been proposed to address combinatorial problems. In the context of continuous optimization, Asghari et al. [40] describe the scientific workflow scheduling problem as a bi-objective optimization problem using a makespan and reliability optimization strategy to resolve workflow scheduling problems from a makespan and reliability perspective. A hybrid bi-objective discrete cuckoo search algorithm (HDCSA) is suggested as a solution to this combinatorial challenge. The hybrid algorithm that is being suggested strikes a fair balance between exploration and exploitation during the optimization process by using various unique Levy flight operators appropriate to discrete search spaces.

Where the consolidation of virtual machines is described as an integer linear programming issue and abstracted to a two-dimensional bin-packing problem. In order to lower the total power consumption (TPC) of the datacenter, Saeedi et al.'s [41] main focus was on the virtual machine (VM) consolidation module, which launches customers' requested VMs on the fewest number of active servers using the discrete optimization. Because resource skewness may force the algorithm to activate more servers, they offer a resource skewness-aware VM consolidation strategy based on an improved thermodynamic simulated annealing (SA) approach.

The management of energy and lifetime maximization of such resource-limited networks remain among the most significant concerns because business continuity in vital industries heavily relies on the network's lifetime. Where The target coverage problem, which calls for each target to be watched by k-different sensors, boosts dependability and promotes fault-tolerant observation in these types of enterprises. If there is significant energy consumption compared to a single coverage issue. In order to escape a local trap, Ekhlas et al. [42] developed the k-coverage challenge for a discrete optimization problem from the perspective of network lifespan expansion and the temperature idea of simulated annealing.

### 2.2 Mocroarray description

Microarray datasets are compilations of genetic data generated using microarray technology and are used in the domains of pharmacy, medicine, clinical research, and physician studies. These databases give important information about the levels of gene expression or genetic variants in biological samples like patient tissues or cells. The significance of each of these datasets in each domain is described below:

Microarray datasets can be used in pharmacy research to examine how different medications or pharmaceutical substances affect cells or tissues. Microarrays are used by researchers to analyze how gene expression profiles change before and after pharmacological treatments. Pharmacologists can design more effective and targeted drugs by studying these databases to find the genes and biological pathways linked to drug reactions. For instance, using microarray data, researchers may examine how cancer cells react to various chemotherapy treatments, resulting in the creation of individualized treatment regimens.

Microarray datasets are used in the medical field to investigate the genetic causes of diseases and comprehend their molecular mechanisms. Comparing the gene expression profiles of healthy and sick tissues is a common task for these datasets. For instance, microarrays in cancer research assist in the identification and categorization of different types of cancer by identifying genes that are elevated or downregulated in tumor cells. The creation of novel treatments and biomarkers for the diagnosis and prognosis of diseases can also be guided by this information.

In clinical research, particularly personalized treatment, microarray datasets are essential. Clinicians can choose the best course of treatment by examining the genetic data of their patients. For instance, using microarray data, oncologists might choose targeted therapy depending on the genetic makeup of a patient's tumor. This strategy can lessen negative effects while improving therapeutic results.

Microarray datasets can be used in medical research to examine the genetic components of particular medical problems or the reactions of patients to therapies. This data can be used by doctors to make data-driven decisions about patient care. Microarrays, for instance, can be used in cardiology to find genetic risk factors for cardiovascular disease and help doctors determine the best preventative measures or treatments for a patient.

The study of microarray datasets in each of these fields calls for sophisticated computational and bioinformatics methods. In order to pinpoint relevant gene expression patterns, pathways, and prospective therapeutic targets, researchers employ a variety of data mining and statistical techniques. A crucial step in translational research, which uses discoveries from the lab to enhance patient care and outcomes, is the integration of clinical and genetic data from microarrays.

Overall, the availability of a wealth of genetic data from microarray datasets has transformed our understanding of diseases, medication responses, and customized medicine. This data can be used to promote research, diagnosis, and treatment in the fields of pharmacy, medicine, clinical practice, and physician studies.

### 2.3 Related works

The theory behind metaheuristic algorithms is to find a particular mathematical strategy that optimizes a specific problem. Improvement is achieved by multiple development efforts appropriate to the problem [43]. Several previous studies have used metaheuristic algorithms to solve the FS problem, including AlFarraj et al. [44], who presented a hyperdization technique using fireflies gravitational and ant colony optimization (FGACO) to pick the optimized functions. A framework for optimizing FS as well as soft computation methods for reducing the dataset's dimensionality. Initially, the data was gathered from a variety of databases, some of which contained inconsistencies, limiting the system's ability to work. The inconsistencies and noise data are then removed using a normalized approach. In addition, Emary and Zawbaa [45] suggested a new version of the ant lion optimizer (ALO) centered on Lèvy fights. The Lèvy antlion optimization (LALO) algorithm is used in this analysis to pick features, and it is evaluated on 21 separate datasets. The findings are assessed using a variety of metrics, including convergence, consistency, feature reduction scale, test data accuracy, and statistical significance, as well as the amount of time it takes to complete the task.

Zhang et al. [46] proposed the two-archive multi-objective artificial bee colony algorithm (TMABC-FS) on several datasets from UCI, which is a multi-objective function discovery approach. It is compared to two regular algorithms and three multi-objective approaches. According to the findings, TMABC-FS is an efficient and stable optimization method for solving cost-sensitive feature selection problems. Further, Ghosh et al. [47] suggest a wrapper-filter hybrid of ACO to minimize computational complexity, in which subset evaluation is added using a filter approach rather than a wrapper method. To execute FS in a multi-objective way, to keep track of the best ants, and to perform dimension-dependent pheromone updates, a memory was used. Using multi-layer perceptron classifiers and K-nearest neighbors In another study, Rao et al. [48] suggested a new FS process based on a bee colony and a gradient-boosting decision tree. With the goal of solving issues such as feature efficiency and informative accuracy, the method achieves global optimization of the decision tree's inputs by using the bee colony algorithm to identify informative characteristics. This method initializes the function space spanned by the dataset. Less essential traits are suppressed using an artificial bee colony algorithm based on the information they bring to decision-making.

Using a hybrid approach, Gao et al. [49] proposed a hybrid cat swarm optimization (HCSO) algorithm for improving the efficiency of simple cat swarm optimization (CSO), which integrates directed, competitive, and implicit features into the actual CSO. The statistical analyses conducted in the experiments show that HCSO has a somewhat greater function selection potential than its competitors. As well, Pandey et al. [50] introduced the binomial cuckoo. The suggested binary binomial cuckoo search algorithm is used to choose the correct subset of features in the dependent hybrid data transformation method, which transforms the initial data first. The method proposed for FS improves classification accuracy while reducing the number of features used.

In more recent studies, Zhang et al. [51] [Binary Differential Evolution of Self-Learning] is a modern multi-objective feature selection approach that is being tested (MOFS-BDE). To boost MOFS efficiency, BDE's three new operations are introduced and integrated. The optimized one-bit filtering Search operator can boost the self-learning capabilities of affective events in optimum areas, and the effective non-dominated filtering operator with flooding distance can reduce the computational difficulty of tagging. Also, Günay and Orman [52] proposed a modified Firefly Algorithm to minimize the data dimension by choosing the features according to the KNN system accuracy values obtained. Through that aspect of the new FFA, memory consumption is significantly decreased by over 50%. Results obtained show this approach saves both time and memory. Also, Vijayanand and Devaraj [53] suggested a modified whale optimization algorithm to create a wrapper-based solution (WOA). The downside of WOA is that it converges too soon, resulting in a local optimal solution. To overcome this limit, the technique that combined genetic algorithm operators with the WOA The whales' search space was increased using the crossover operator, and the local optimum was avoided using the mutation operator. The proposed method extracts useful features from network data, making intrusion detection more efficient. The types of intrusions were described using SVM based on the chosen characteristics.

In another piece of research, Marie-Sainte and Alalyani [54] proposed a modern approach to feature discovery based on the Firefly algorithm. A host of combinatorial problems have been solved using this algorithm. However, it has not been interested in the principle of FS for Arabic text classification. This approach is checked using the Support Vector Machine classifier as well as three calculation steps such as precision, recall, and F-measure. As well, Zhu et al. [55] proposed an improved version of the gravitational search algorithm called IGSA; the idea of global memory is added, and the definition of exponential K is used. The location of the best solution found so far is memorized in this algorithm, which can be useful. Preventing ions from clumping together and moving slowly. As a result, the algorithm's extraction capacity improves, and a balanced approach to exploration and exploitation is created. Furthermore, the exponential K will reduce the running time considerably. A binary IGSA (BIGSA) is also implemented to solve the FS problem.

Furthermore, Han et al. [56] suggested a two-phase FS technique for cancer classification. By combining ReliefF with a recursive binary gravitational search algorithm, this approach selects a low-dimensional set of genes to identify biological samples of binary and multi-class cancers (RBGSA). At each recursive point of the algorithm, the proposed RBGSA refines the gene space from a coarse to a fine-grained level without losing precision. In another work, Faris et al. [57] suggested an improved binary salp swarm algorithm (SSA) to create an improved FS wrapper method as a search technique, along with a RWN classifier as an induction algorithm. It replaced the original static approach with a dynamic strategy to monitor the number of leaders and followers.

Using a genetic algorithm (GA), Rostami et al. [58] suggested a method based on community detection that operates in three steps. In the first step, feature similarities are determined; in the second, category recognition algorithms categorize the features into clusters. In the third stage, a genetic algorithm selects features with a modern community-based repair mechanism. Also, Mehrdad et al. [59] proposed a community detection-based GA. It works in three stages. The authors contrasted the performance of the proposed solution with the results of four existing FS algorithms. Three experimental FS approaches based on PSO, ACO, and ABC algorithms were reviewed and compared. The results show that it has better accuracy, faster convergence, and better search quality.

In a recent study, Alweshah et al. [15] proposed dual methods, the first of which is the mine blast algorithm MBA. The second is MBA with simulated annealing to decrease the feature numbers selected to increase the accuracy rate. While it can substantially reduce the number of functions, it cannot accomplish high precision compared to other methods. In addition, Alweshah et al. [60] implement the monarch butterfly optimization algorithm to solve FS problems. The MBO algorithm is a population-based swarm intelligence algorithm that uses a swarm intelligence approach. The total number of butterflies in the population can be found in either Land 1 (the pre-migration home) or Land 2 (the post-migration home). Use the monarch butterfly optimization algorithm to solve FS problems. The total number of butterflies in the population can be found in either Land 1 (the pre-migration home) or Land 2 (the post-migration home).

For Gene Selection Problems, Khurma et al. [61] proposed a hybrid MH-MH wrapper called IMFOHHO for the GS problem. To improve the exploration and exploitation phases, the proposed approach combines the Moth Flame Optimization (MFO) and Harris Hawks Optimization (HHO) algorithms. The main goal is to combine the best features of both algorithms into a single model and overcome their shortcomings in the gene search space. Combining two swarm systems into one model allows for thorough gene space exploration and a wide range of solutions. Ten gene expression data sets were used to evaluate the proposed method's performance. The comparison study shows that the IMFOHHO model improves classification performance without adding to the computational load.

In another study on gene selection, Almugren and Alshamlan [62] applied the gene expression datasets to the genetic algorithm GA, which achieves the highest accuracy with relatively small numbers of selected genes. As well, Alshamlan [63] proposed Co-ABC, the Correlation-based Artificial Bee Colony algorithm, a modern hybrid approach focused on FS and for selecting a limited number of appropriate genes for correct gene expression feature classification. The experiments show that using a tiny proportion of diagnostic genes, the proposed Co-ABC algorithm achieves accurate classification. Furthermore, Pirgazi et al. [64] suggest a hybrid approach based on the IWSSr method and the Shuffled Frog Leaping Algorithm (SFLA) to pick successful features in a large gene dataset. The method is tested using several normal gene expression datasets. The experimental findings show that the suggested solution, in contrast to similar approaches, has a more compact range of features along with high precision.

As mentioned, FS is a difficult problem that necessitates the use of intelligent dataset-handling methods in order to select the appropriate attributes for the research problem. Following the submission of literature, a variety of algorithms are used to solve the problem. The majority of these methods have been used multiple times, either alone or in combination with other algorithms, or by refining the same algorithm to provide good methods in this area. Furthermore, it is self-evident that no one solution can be used to solve any type of problem effectively because no approach is best in all cases and each method has advantages in different fields of interest, with outcomes varying depending on the type and complexity of the problem. Also, gene expression results are often repetitive and noisy, with just a small percentage of them presenting different profiles for various types of samples. As a result, in the field of bioinformatics, It's becoming more and more interesting to select highly discriminative genes from gene expression data.

Thus, in this study, a new approach is applied to reduce the features of gene expression that are often repetitive and noisy and to produce resolutions with more reliable results in a sensible period of time. The proposed approach is based on the use of the BWO, which is one of the latest metaheuristic algorithms. It has been used in various applications and demonstrates its ability to deal with a variety of problems in various fields of engineering optimization, for example, document clustering, cloud computing optimization, and information retrieval. As well, it has the capability to deal with the challenges of real-world optimization, the ability to have a high degree of convergence, and the ability to offer a high-quality performance [[65], [66], [67], [68], [69], [70], [71], [72]]. For that, BWO was applied for GS in two ways: it was employed in its original form, and it was hybridized with an iterated greedy (IG) algorithm, BWO-IG, to improve the BWO's local search capabilities.

## 3 Black widow optimization algorithm

Hayyolalam and Kazem [32] introduced BWO, which is a Metaheuristic algorithm that was recently created. The fundamental concept of this algorithm is that the odd mating behavior of BWO inspired the creation of a new Metaheuristic optimization algorithm. The key inspiration for the invention of this new algorithm was the unique feature of black widows marrying and producing new generations. Some of the most critical works completed using this algorithm will be discussed in this section, as well as how this algorithm led to the desired outcomes [73].

There are four main stages in BWO: initialization, procreation, cannibalism, and mutation. The procreate stage emphasizes the discovery of the quest domain by producing many offspring. Then, by omitting incorrect solutions in the cannibalism stage, the BWO algorithm can progress quickly toward the right solution. The mutation process ensures that the exploitation and discovery phases are performed in the correct order [74].

The first step is to customize the BWO algorithm's parameters. Then, the next step is to create the initial population, which defines the GS problem's candidate solutions (widows). To increase the diversity among the widows, the created population is then updated using a crossover and mutation relational algorithm. The fitness values of the widows are calculated next, and the best widow is determined. Then the BWO upgrades the widow based on the fitness function's consistency (measured by its probability). The BWO is used if the probability of the fitness function for the new widow is greater than 0.5 [65]. The fitness function possibility is calculated by Eq. (1). The BWO algorithm updates the new widow based on the importance of Proi.

(1) $$Proi=\frac{fi}{\sum_{i=1}^{n}fi}$$

Then, the best widow is chosen from the updated population. Following that. If the stopping condition is satisfied, the best gene subset is evaluated on the gene datasets (external evaluation). The steps from calculating the chance of the new widow to the end are replicated if the stopping condition is not satisfied. After a certain number of loops, the algorithm comes to a halt and outputs the best gene subset that has been discovered. The finest gene subset is then produced as the preferred set of genes for use in the classification system's architecture [75].

Spiders (order Araneae) are air-breathing arthropods with eight legs and venomous fangs on their chelicerae (in the jaw area). Spiders constitute the largest order of arachnids, and they rank seventh among all animals in terms of species diversity [76]. The basic idea of BWO is inspired by the unusual mating behavior of one species of spider, namely, the black widow spider. The BWO approach includes a one-of-a-kind stage known as cannibalism, whereby individuals of the species that are of insufficient fitness are excluded from the circle, which leads to early convergence [66].

The female black widow is mostly nocturnal, remaining hidden throughout the day and spinning her web at night. A female widow, in most cases, spends the majority of her adult life in the same place. A female black widow attracts a male by spraying pheromone on certain parts of her net. By minimizing the size of their web, the first male to enter the web decreases the attraction of females to rivals. The female eats the male during or after mating, then moves the eggs to her egg sock. The offspring engage in sibling cannibalism after hatching from their eggs. They also remain on their mother's web for a limited time, and they can even eat the mother. Fit and strong individuals survive as a result of this cycle. Modanu et al. [77].

The male black widow spider is one of two known creatures that deliberately assists the female in sexual cannibalism. During the mating procedure, the female consumes the male fully in only two out of every three instances. Males that are not eaten succumb to their injuries immediately after mating [78].

The BWO algorithm starts with a population of spiders, each representing a potential solution. In pairs, the first spiders try to reproduce the new generation. The male black widow is consumed by the female black widow during or after mating. She then transports and releases the sperm she has stored in her sperm thecae to the egg sacs. Spiderlings can emerge from egg sacs up to 11 days after they have been laid. They cohabitate on the maternal web for a few days to a week, during which time sibling cannibalism occurs. They are then swept away by the storm [67].

### 3.1 Steps of the black widow optimization algorithm

#### 3.1.1 Initialization of population

In order to solve an optimization problem, the values of the problem variables must form an appropriate basis for solving the current problem. This arrangement is referred to as a “chromosome” and a “particle position” in GA and PSO terminology, respectively, and as a “widow” in the BWO context. The potential solution to each problem is modeled after a black widow spider. The values of the problem variables can be seen on these spiders. Hence, when. When solving benchmark functions, the structure can be seen as an array.

A widow is exemplified as an array of 1 × Nvar, signifying the problem solution as a Nvar-dimensional optimization issue, and is defined as per Eq. (2) below:

(2) $$Window=\left(x_{1},x_{2},.\;.\;.\;.\;.\;.\;.\;.\;x_{Nvar}\right)$$

Each variable value *(x1, x2, …, xN*_*var*_*)* indicates the number of floating points. A widow's fitness is achieved by testing its fitness model *f (*$x_{1},x_{2},.\;.\;.\;.\;.\;.\;.\;.\;x_{nvar}$*)* by Eq. (3).

(3) $$Fitness=f\left(window\right)=f\left(x_{1},x_{2},.\;.\;.\;.\;.\;.\;.\;.\;x_{nvar}\right)$$

To begin the optimization algorithm, a candidate widow matrix of size *(Npop, Nvar)* is generated with an initial population of spiders. The male black widow is eaten by the female black widow before or during mating, and pairs of parents are selected at random to carry out the procreation stage of mating.

#### 3.1.2 Procreation

As the pairs are self-contained, they begin mating in order to reproduce the new generation in tandem, as they do in nature. Regardless of the others, each pair has its own network. In the real world, each mating releases about 1000 eggs, but some spider babies live and grow up to be larger. Now, in order for this algorithm to reproduce, an array called alpha should be created that is as long as the widow array and includes random numbers, and offspring should be created by using Eq. (4), where ×1 and ×2 are parents and y1 and y2 are offspring:

(4) $$alpha=\left\{ \begin{array}{c} y_{1=\alpha \times x_{1+\left(1-\alpha \right)\times x_{2}}}\\ y_{2=\alpha \times x_{2+\left(1-\alpha \right)\times x_{1}}} \end{array}\right.$$

This procedure is replicated Nvar/2 times, with the exception that the numbers chosen at random cannot be duplicated. The children and their mother are then added to an array and sorted by fitness value; some of the best ones are then added to the newly formed population based on their cannibalism ratings.

#### 3.1.3 Cannibalism

There are three different types of cannibalism in this scenario. The first is sexual cannibalism, in which a female black widow consumes the male during or after mating. Note that it is possible to distinguish between female and male fitness values using the BWO algorithm. Another type of cannibalism is sibling cannibalism, in which stronger spiderlings kill their weaker siblings. In this algorithm, a cannibalism rate (CR) is set, which determines the number of survivors. The third type of cannibalism is when baby spiders eat their mothers. The fitness value is used to determine how strong or frail the spiderlings are.

#### 3.1.4 Mutation

**Fig. 1:**
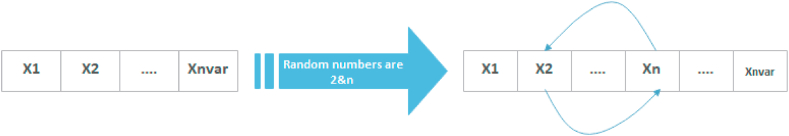
Mutation.

In the mutation stage, a mutepop number of individuals are chosen from the population. As seen in Fig. 1, each of the selected solutions swaps two elements in the sequence at random. The mutation rate is used to measure the mutepop.

#### 3.1.5 Convergence

In BWO, as in most evolutionary algorithms, there are three stopping criteria that are considered for convergence: (a) the number of iterations, which is predetermined; (b) the discovery of no difference in the strongest widow's fitness value over many iterations; and (c) attainment of the appropriate level of precision.

#### 3.1.6 Parameters setting

To boost the algorithm's performance in finding superior solutions, the parameters should be modified accordingly. The higher the risk of leaping out of any local optimum and the greater the potential to explore the quest space internationally, the more parameters can be fine-tuned. As a result, the correct number of parameters will ensure a good balance between exploitation and exploration. The BWO algorithm has three important control parameters: the procreation percentage (PP), cannibalism rate (CR), and mutation rate, which are defined as follows.

• The PP specifies how many individuals can take part in the procreation stage. This parameter, by regulating the development of different offspring, contributes to diversification and allows for more detailed exploration of the search space.
• The CR excludes unacceptable individuals from the community. By switching search investigators from local to global search and vice versa, the right value for this parameter enables great efficiency in the exploitation stage.
• The PM indicates the percentage of the population that is undergoing mutation. This parameter's right value ensures that such exploitation and exploration stages are balanced. This parameter can be used to monitor the progress of the search agents as they move from the global to the local level and can also be used to guide them to the best solution.

The BWO algorithm's general pseudocode is shown below.

**Algorithm 1:** Pseudocode of BWO Algorithm

**1. Setting the parameters-**2. Population size, *max*Iteration, number of features, procreates rate and mutation rate. 3. Generating a random sample of solutions for the original population. 4. Finding the fitness value to each solution based on Eq (1). 5. Assess all obtained solutions and save them in (pop1) depending on their fitness score. 6. Assign W* to the population's best solution. 7.7. Determine the number of replicas (*Nr*) based on Pr. 8. Determine the number of mutations (*Nm*) based on *M*r. 9. Let x = 0 **10. While** x < *max*Iteration **Then** **11. for** x = 1 to (*Nr* ÷ 2) 12. Choose two solutions ×1 and ×2 at random as parents from pop1. 13. Using Eq. (4), create two children, y1 and y2. 14. Using Eq. (1), Find the fitness values of y1 and y2. 15. Depending on their fitness rating, terminate the father ×1 or x2. 16. Depending on their fitness rating, remove one of the two children y1 or y2. 17. Save the rest of the solutions to pop2. **18. end for 19. for** x = 1 to (*Nm*) 20. Choose a solution from pop1 at random. 21. Use the mutation mechanism on the chosen solution. 22. Save the new solution in pop3. **23. end for** 24. Modify the population to be pop2+pop3. 25. Using Eq. (1), assess all solutions inside the population. 26. Update W*.

## 4 Iterated greedy algorithm

A stochastic search approach, the iterated greedy algorithm (IG), is used in this study. IG reconstructs a full candidate solution by iterating across greedy, constructive heuristics that build a succession of solutions. An acceptance criterion determines whether the newly developed solution will replace the original solution when it has been completed. IG repeats these actions until a stop requirement is reached. Iterative Local Search (ILS) is comparable to IG, except that instead of iterating more than a local search like ILS does, IG iterates over construction heuristics [79].

During the destruction process, a simple random sample of solution genes d is chosen at random and removed from the solution without repetition, yielding two partial solutions. It is first delivered as SR, with only a size d of genes, as well as the genes that were deleted in the same order that they were removed. The original final solution, with size n-d of genes, has been donated as SD and does not contain any of the deleted genes [80].

The reintroduction of genes that have already been eliminated from the solution occurs during the building process. The NEH insertion heuristic has been used as a constructive strategy to complete the answer. The first gene, SR1, gets put into all accessible n-d+1 sites in the destroyed solution SD, culminating in n-d+1 obtaining a solution. Only the n-d+1 partial solution that are better is picked and saved for the next iteration. The technique is repeated till the SR is exhausted or the ultimate solution is found, at which time the second gene is investigated. Once again, SD has a size of n [13,80].

Following the demolition and construction stages, we then decide if the new sequence is acceptable as the incumbent solution for the following iteration. Accepting new sequences if only they produce a higher value is one of the basic success criteria. However, because of insufficient diversification, an IG algorithm utilizing this acceptance criterion may result in search stagnation. Then, a basic simulated annealing-like acceptance criterion with a constant temperature (similar to that criterion in Ref. [81]) was used in this paper. This constant temperature is calculated based on Eq. (5).

(5) $$T=\frac{\sum_{i=1}^{n}\sum_{j=1}^{m}P_{ij}}{n*m*10}$$

where a new solution is only examined to see whether it is better than the others, the worst is approved with a probability of being the worst. One difference would be that the temperature is constant here, meaning there is no cooling method. The IG pseudocode is used in Algorithm 2.

**Algorithm 2:** Pseudo-Code of IG algorithm

1. Create the first solution x. 2. Setting the beginning temperature T to be T0 **3. for i** = **1 to (d)** 4. Delete one gene from x at random and put it to ×2, ×2 would be a second array that stores the genes that were removed from x. **5. End for 6. for i** = **1 to (d)** 7. Let $x_{0}$ to be the best permutation that can be produced by adding a gene $x_{i}$ in any possible position of x **8. End for** 9. If the quality of $x_{0}$ is better than the x then 10. x = $x_{0}$ 11. Else 12. Let 1E equal f ($x_{0}$) − f (x) 13. Generating a random (r) between 0 and 1 14. If (r < e −1E T) then 15. Let x equal $x_{0}$ 16. End if 17. End if 18. Modify the best solution 19. Return best solution.

## 5 Proposed approaches

In the FS generation step, a collection of attributes is chosen from the entire dataset and subjected to an evaluation process to determine whether it matches the solution or not. Forward selection or backward exclusion may be used in this process. Both methods perform an exhaustive search of the solution space. A full search means that no optimum subset is skipped, but it takes a long time to compute such a search because the GS problem is massive.

However, there is another way to extrapolate subsets that is based on random sampling. This type of method can be useful for the FS process in that it reduces the need to wait before the search is done because an iteration value is used to complete the search. And despite the fact that the random process reduces calculation time, it is not always the best option. In the FS process, a metaheuristic algorithm can be used to randomly categorize subsets. It gathers data during the hunt to help guide the process and then creates new solutions by integrating one or more good solutions.

Feature selection is regarded as a binary optimization problem, where solutions are restricted to binary numbers (0, 1). When using BWO for FS problems, this means that any optimization strategy that is used to solve FS problems must be converted into a binary version, so that the solutions are expressed as 0 and 1 in a one-dimensional vector, where the value of 1 reflects that the attribute is chosen; otherwise, the value would be (0).

**Fig. 2:**
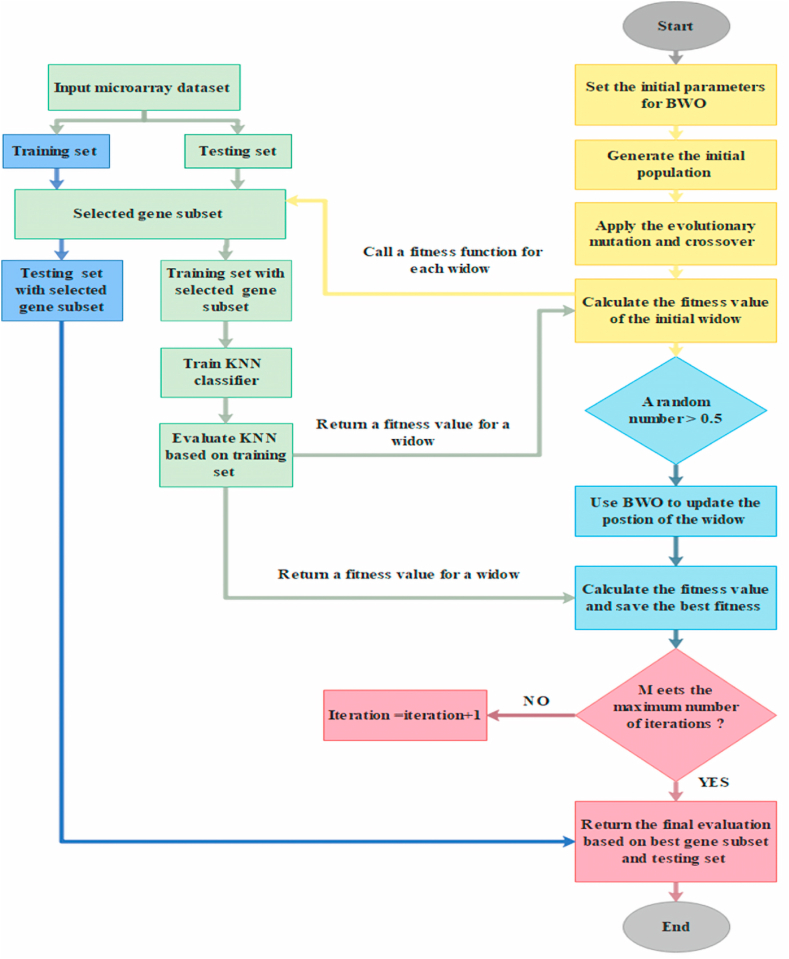
Flowchart of proposed BWO method.

In this study, the K-nearest neighbor KNN classifier is used as a wrapper FS method [82]. The KNN classifier dictates the accuracy rate of the BWO's algorithm's FS process. According to the literature, the KNN has been shown to have high classification efficiency in FS problems. Fig. 2 shows the proposed BWO for gene selection.

In the current study, two models are proposed. To choose the most important features from a dataset, both models employ the wrapper FS technique. The first is based on the basic BWO, while the second combines the BWO with the IG to establish a balance in the BWO between exploration and exploitation.

More specifically, the present research posits a gradual hybridization of the BWO and IG. During the hybridization procedure, the IG is incorporated into the BWO improvement process. The hybridization process starts with a certain number of BWO repeats. The IG obtains the best solution and greatest fitness found by the BWO after the required number of iterations and begins its improvement process. The IG then submits the obvious solution and fit that it identifies to the BWO to continue the development process. This reciprocal operation is repeated once all of the BWO iterations are complete and the halting condition is met.

**Fig. 3:**
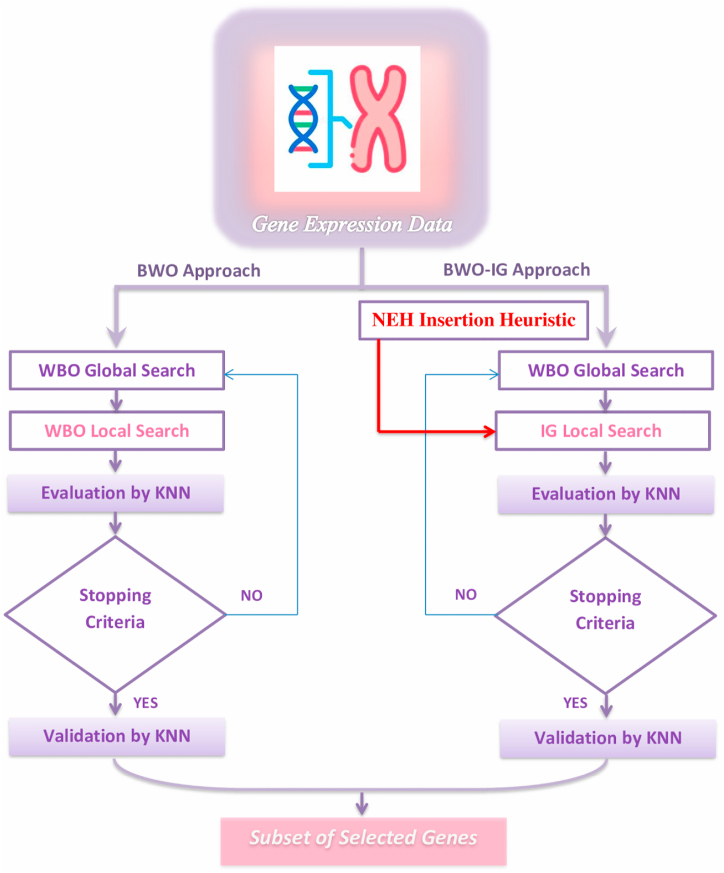
Proposed a wrapper BWO and BWO-IG for gene selection.

The proposed BWO-IG technique employs BWO to generate the initial population of viable solutions. In the second step, the IG analyzes the fitness value of all candidates to discover better solutions, guaranteeing efficient convergence and high-quality solutions and eventually reaching the optimal parameter values, enhancing classification accuracy. The recommended BWO-IG with KNN solution to the FS issue is shown in Fig. 3.

As is known, the method of choosing attributes is determined by two factors: the degree of precision and the number of features chosen. The fitness feature was created to assess the efficiency of the process in achieving a balance between these two factors. The fitness function criterion used is described by Eq. (6).

(6) $$Fitness=\alpha \gamma R\left(D\right)+\beta \frac{|R|}{|N|}$$

where γR(D) is the classification error rate obtained by KNN, R is the number of features in the chosen subset, N refers to the number of features in the original dataset, and two parameters α and β correspond to the significance of classification consistency and subset length respectively, where α takes values between 0 and 1; α ∈ [0, 1], and β = (1–α) [83].

## 6 Scope of the problem

After the explanation of the problem, the initial step before building the methodology is to define the problem domain, and this is done via a mixture of means to accumulate the data that is related to the problem.

### 6.1 Datasets used

In this section, we present the datasets that will be used in experiments. Nine microarray datasets are applied to test the efficiency of the intended method. The used datasets are MLL, Colon, Leukemia, Leukemia_c3, Leukemia_c4, CNS, Ovarian, SRBCT, and lymphoma. These data sets, which include small, medium, and high-dimensional data sets, are widely used in many experiments. Table 1 summarizes the features of the chosen data sets.

Experiments were carried out on nine benchmark datasets from the gene expression data warehouse [2] to assess the efficiency of the proposed methods. The number of genes and sampels in each of these datasets are mentioned in Table 1 belew.

**Table 1:** Gene expression datasets characteristics.

Dataset	No. of Genes	No. of Samples	No. of Classes
**MLL**	12,582	72	3
**Colon**	2000	62	2
**Leukemia**	7129	72	2
**Leukemia _c3**	7129	72	3
**Leukemia_c4**	7129	72	4
**CNS**	7129	60	2
**Ovarian**	15,154	253	2
**SRBCT**	2308	83	4
**lymphoma**	4026	62	3

### 6.2 Description of the datasets

#### 6.2.1 MLL

A class of human acute lymphoblastic leukemias known as mixed-lineage leukemias (MLL) have a chromosomal translocation affecting the mashed leukemia gene. MLL transposable elements are most frequent in babies and chemotherapy-induced leukemias, and they have an especially bad diagnosis. 24 examples of acute lymphoblastic leukemia (ALL) diagnosis groups (33.3%) MLL (mixed-lineage leukemia): 20 case reports (27.8%) 28 cases of acute myeloid leukemia (AML) (38.9%).

#### 6.2.2 Colon

This dataset is identical to the yeast gene expression dataset in that it includes expression amounts for 2000 genes from 62 samples. It is noted whether each sample came from a tumor surgery or not. It can be used in two ways: the 62 samples can be treated as records in a high-dimensional vacuum, or the genes can be treated as records with 62 attributes.

#### 6.2.3 Leukemia

The representation levels of 7129 genes were measured in 72 samples for the leukemia data collection. The labels show which of two types of leukemia were found in the study: AML with 25 samples or ALL with 47 samples. Since this dataset is similar to the colon cancer dataset, it should be included in the same studies.

#### 6.2.4 CNS

Central Nervous System There are 60 clinical samples in the data set. There are 21 winners (labeled “Class1″) and 39 defeats (labeled “Class2″) (labeled “Class0″). There are 7129 genes in the dataset.

#### 6.2.5 Ovarian

This dataset incorporates information from four randomized double-blind clinical trials in advanced ovarian cancer (Ovarian Cancer Meta-Analysis Project, 1991). The aim of these studies was to compare the efficacy of cyclophosphamide plus cisplatin (CP) versus cyclophosphamide plus adriamycin plus cisplatin (CAP) for advanced ovarian cancer treatment.

#### 6.2.6 SRBCT

The small round blue cell tumors (SRBCTs) are a group of four childhood tumors that have a common existence on regular histopathologic examinations, making accurate doctor's manifestations difficult. Even so, correct diagnosis is important because medical therapy, medication reactions, and prognoses differ considerably on the basis of the diagnosis Several among these are Ewing's tumors (EWS), neuroblastoma (NB), non-Hodgkin lymphoma (in our case, Burkitt's lymphoma, BL), and rhabdomyosarcoma (RMS).

#### 6.2.7 Lymphoma

The lymphoma dataset includes 42 samples of diffuse large B-cell lymphoma (DLBCL), 9 samples of follicular lymphoma (FL), and 11 samples of chronic lymphocytic leukemia (CLL). The DBLCL, FL, and CLL classes are coded as 0, 1, and 2 in the y vector, respectively. Data on gene expression was normalized, imputed, log converted, and standardized through genes to a zero mean and unit variance.

## 7 Experimental results

In this section, the proposed approaches, BWO-IG and BWO, were evaluated using the nine gene expression datasets to determine their efficiency for GS problems, and the findings were then compared with the results of other methods in the literature. All Experiments on all datasets were executed using Matlab R2015b on an Intel ® CoreTM i5 8th generation CPU with 12 GB RAM and, on a Windows 10 operating system.

### 7.1 Evaluation scenario and comparative works

A thorough examination covering all aspects of our study considerably enhanced the evaluation process. In order to begin this study, we compared two fundamental versions, BWO and BWO-IG, primarily in terms of their classification precision and sample size. In Table 3, these perceptive comparisons have been painstakingly collated to give a clear picture of how they performed.

Examining the statistical elements, wherein we compared BWO with BWO-IG, gave our opinion more depth. This required a thorough analysis of the statistics and related p-values obtained from the T-test for accuracy, which is beautifully displayed in Table 4.

**Fig. 4:**
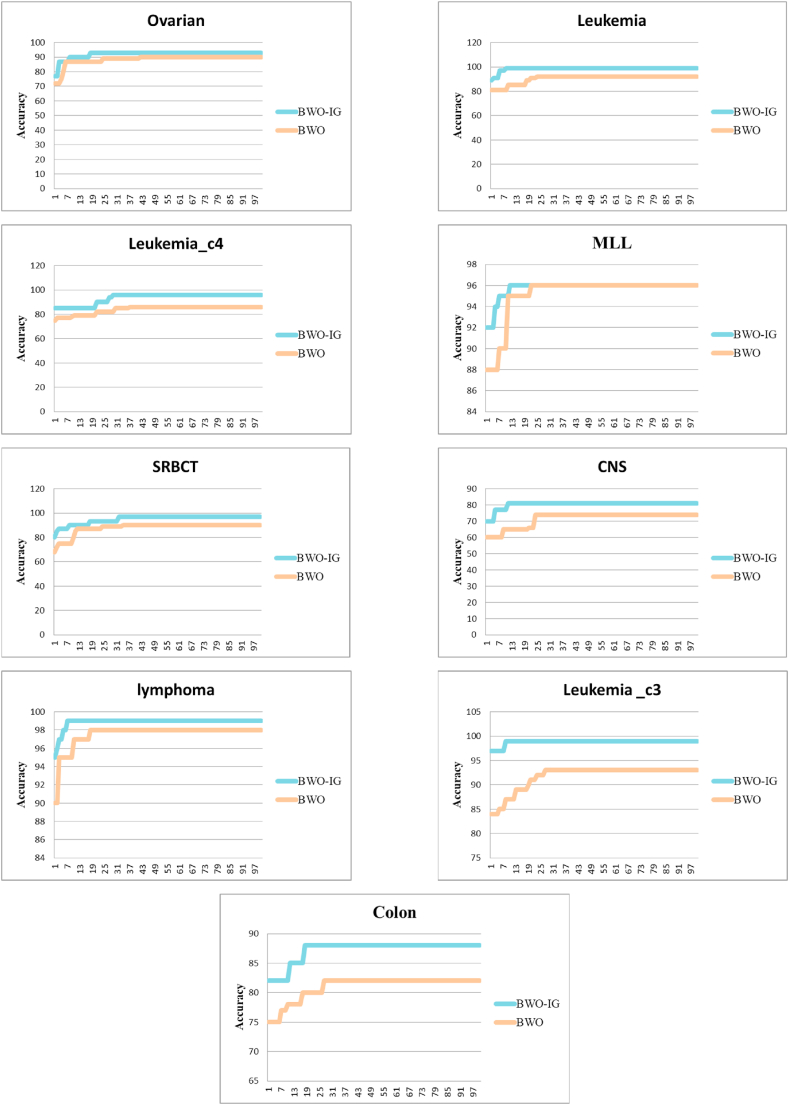
Convergence speed of BWO and BWO-IG.

We scrutinized the convergence speed of BWO and BWO-IG to acquire a comprehensive grasp of their behavior, which is skillfully depicted in Fig. 4. This visual illustration provides a dynamic viewpoint on each optimization trajectory.

We next compared the classification accuracy of BWO-IG to the most advanced optimization techniques in an effort to put our results into context. Table 5 contains the meticulously detailed results of this thorough analysis, which provide a reference point for our comprehension of the relative performance of BWO-IG.

**Fig. 5:**
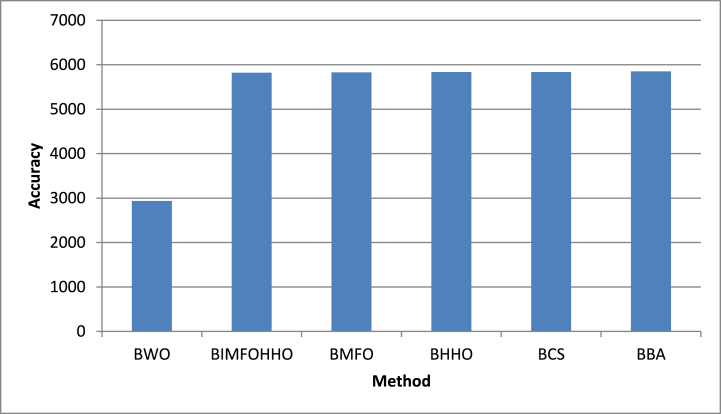
Classification accuracy of all methods.

We combined all of the techniques used in our investigation in terms of classification accuracy to provide a more comprehensive view of the landscape of our study, which is beautifully represented in Fig. 5. This graphical display provides a broad overview of how our suggested strategies stack up against rivals.

We also investigated the performance of BWO-IG in comparison to cutting-edge optimizers, concentrating on their average fitness values, which are neatly arranged in Table 6. This comparison sheds important light on the BWO-IG's optimization effectiveness.

Additionally, we compared the average number of genes chosen, a key component of our study, between our suggested procedures and cutting-edge techniques. Table 7 contains this insightful analysis, which highlights the diverse gene selection capacities of various strategies.

**Fig. 6:**
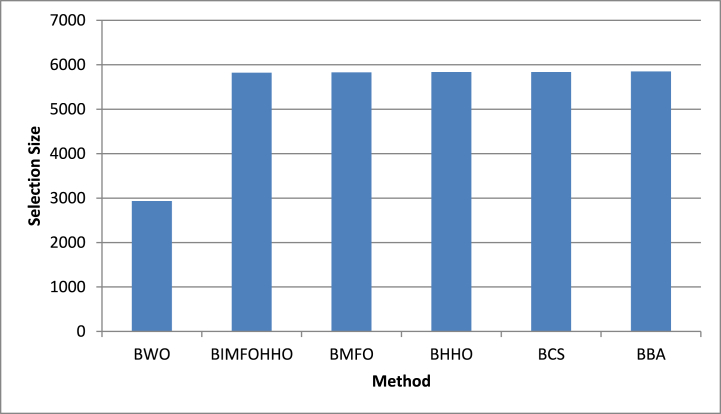
Number of genes selected by all approaches.

The interaction between the number of genes chosen by each method and their corresponding average fitness values was our final focus. Fig. 6 beautifully captures this interaction. The complex relationship between gene selection and optimization efficiency is captured in this image.

In conclusion, our review process covered a wide range of analyses, offering light on different aspects of our study's contributions and the relative effectiveness of the methodologies under discussion.

### 7.2 Parameter settings

The wrapper FS model is used in the proposed method for the evaluation process, while the BWO and IG algorithms are used to find the (near) optimal function subset. A KNN classifier is used to classify the data, and K = 5 is used as an evaluator to rate the quality of the function subsets chosen. Peterson [84], K-fold cross-validation is used to split each dataset into training and testing datasets. K-1 folds are used for preparation and validation, and the remaining fold is used for research in this technique. The input parameters for all experiments performed on all datasets were based on extended experimental trials that showed that the proposed method performs well with certain parameter values. Table 2 shows the parameter settings.

**Table 2:** Input parameter settings.

Parameter	Value
**UB (upper bound)**	1
**LB (lower bound)**	0
**d-max (max distance)**	Le-2
**Number of iterations**	100
**Population size**	10
**K (for KNN classifier)**	5
**pr**	0.6
**mr**	0.4

### 7.3 Results for classification quality

This section presents details of the classification accuracy that was achieved by applying the BWO-IG and BWO methods to each dataset. The results are presented in Table 3. As seen, the BWO-IG approach surpassed the original BWO in all datasets, either in classification accuracy or in the number of selected features.

**Table 3:** Classification accuracy and selection size of BWO and BWO-IG.

Dataset	Method	Classification Acuracy	Selection size
**MLL**	BWO-IG	96.2030	33
	BWO	96.0300	35
**Colon**	BWO-IG	88.2790	22
	BWO	0.80530	24
**Leukemia**	BWO-IG	99.0740	30
	BWO	0.92600	32
**Leukemia _c3**	BWO-IG	98.8880	33
	BWO	0.93200	40
**Leukemia_c4**	BWO-IG	96.1110	29
	BWO	0.87400	34
**CNS**	BWO-IG	81.6660	28
	BWO	0.74900	36
**Ovarian**	BWO-IG	92.5450	116
	BWO	0.90330	132
**SRBCT**	BWO-IG	97.1930	30
	BWO	0.90830	39
**lymphoma**	BWO-IG	99.8830	29
	BWO	0.98770	41

As shown in Table 3 and in all datasets, the BWO-IG method surpassed the BWO in terms of accuracy and selection size. This means that if the BWO's search mechanism is altered, it will be able to produce more reliable results.

Also, a T-test was performed to evaluate the efficacy of the BWO and BWO-IG methods when applied to the nine datasets. The findings statistics are calculated using the stated approaches, which are dependent on the correctness of the results particular to each dataset. Table 4 shows the results of a T-test on the p-values and classification accuracy obtained by the BWO and BWO-IG with a 95% confidence interval (alpha = 0.05) on the p-values and classification accuracy generated by the BWO and BWO-IG.

**Table 4:** Statistics and P-values of T-test for accuracy of BWO and BWO-IG.

Dataset	Methods	Mean	Std. deviation	Std. error mean	P-value
**MLL**	BWO-IG	0.96	0.02870	0.00524	0.00
	BWO	0.96	0.01474	0.00269	
**Colon**	BWO-IG	0.88	0.04521	0.00825	0.00
	BWO	0.80	0.05557	0.01015	
**Leukemia**	BWO-IG	0.99	0.01685	0.00308	0.00
	BWO	0.92	0.06667	0.01217	
**Leukemia _c3**	BWO-IG	0.98	0.02485	0.00454	0.00
	BWO	0.93	0.03022	0.00552	
**Leukemia_c4**	BWO-IG	0.96	0.02881	0.00526	0.00
	BWO	0.87	0.02673	0.00488	
**CNS**	BWO-IG	0.81	0.06112	0.01116	0.00
	BWO	0.74	0.04088	0.00746	
**Ovarian**	BWO-IG	0.92	0.02470	0.00451	0.00
	BWO	0.90	0.03854	0.00704	
**SRBCT**	BWO-IG	0.97	0.03172	0.00579	0.00
	BWO	0.90	0.02743	0.00501	
**lymphoma**	BWO-IG	0.99	0.00553	0.00101	0.00
	BWO	0.98	0.01736	0.00317	

Table 4 shows that the BWO-IG is somewhat more efficient than the original BWO, with a total of fewer than 0.0001 p-values for the 24 datasets. These findings suggest that the BWO-IG is effective in addressing GS problems.

It is widely accepted that superior solutions result from a steady and rapid rate of convergence. Therefore, one of the goals of this study was to speed up convergence by reducing randomization and the duration of the discovery process, thereby finding the best solution in a shorter amount of time. The BWO and BWO-IG can efficiently find optimal solutions for the nine datasets within a quick convergence time. Fig. 4 depicts the convergence times of the two suggested approaches over 100 iterations when applied to each of the nine datasets.

Fig. 4 shows that the BWO-IG was able to improve classification accuracy while reducing convergence time compared to the BWO. This was accomplished by the GDA enhancing the original BWO's worldwide search capabilities. As seen in Fig. 4, in all datasets, the BWO-IG reaches the best solution in less than 30 iterations. These promising results are due to the ability of the hybridization of BWO-IG to strike a good balance between global and local search while looking for solutions, which prevents the search process from becoming stuck in local optima.

### 7.4 Comparison with previous methods

In this section, a comparison is made between BWO-IG and five methods in the literature, which are Binary Improved Moth Flame Optimization and Harris Hawks Optimization (BIMFOHHO) KHURMA et al. [61], Binary Moth Flame Optimization (BMFO) Khurma et al. [85], and Binary Harris Hawks Optimization (BHHO) Thaher et al. [86]. As well as the BBA and BCS, which are two well-known SI algorithms that are used as wrapper-GS approaches. Table 5 and Fig. 5 show the results for the comparison of the BWO-IG and the other methods in terms of classification accuracy. The best results are shown in bold in the table.

**Table 5:** The comparison of the BWO-IG and the other methods in terms of classification accuracy.

Dataset	BWO-IG	BIMFOHHO	BMFO	BHHO	BCS	BBA
**MLL**	**96.203**	95.403	95.341	95.331	95.318	95.306
**Colon**	**88.279**	79.422	79.391	79.395	79.395	79.303
**Leukemia**	**99.074**	85.846	85.822	85.830	85.830	85.202
**Leukemia _c3**	**98.888**	83.155	83.140	83.153	83.153	83.149
**Leukemia_c4**	**96.111**	81.443	81.425	81.450	81.431	81.434
**CNS**	**81.666**	66.510	66.456	66.487	66.487	66.432
**Ovarian**	**92.545**	91.648	91.623	91.647	91.647	91.604
**SRBCT**	**97.193**	87.640	87.623	87.628	87.628	87.623
**Lymphoma**	**99.883**	98.321	98.315	98.325	98.416	98.323
Average	94.426	84.819	84.443	84.500	82.9557	82.863

As seen, the BWO-IG outperformed all five methods in terms of accuracy in all nine datasets. Furthermore, Table 6 shows the comparison of the Average Fitness Values of BWO-IG and Other optimizers.

**Table 6:** Comparison of the average fitness values of BWO-IG and other optimizers.

Dataset	BWO-IG	BIMFOHHO	BMFO	BHHO	BCS	BBA
**MLL**	**0.041**	0.954	0.997	0.972	0.998	0.999
**Colon**	0.145	**0.101**	0.220	0.203	0.159	0.237
**Leukemia**	0.013	0.127	0.171	**0.009**	0.186	0.474
**Leukemia _c3**	**0.015**	0.337	0.337	0.252	0.295	0.315
**Leukemia_c4**	**0.042**	0.227	0.337	0.111	0.295	0.229
**CNS**	0.185	0.202	0.205	0.158	**0.068**	0.312
**Ovarian**	**0.078**	**0.125**	0.171	0.158	0.242	0.198
**SRBCT**	**0.030**	0.328	0.343	0.330	0.339	0.343
**Lymphoma**	**0.004**	0.174	0.187	0.150	0.013	0.175
Average	**0.061**	0.281	0.349	0.299	0.329	0.399

As shown, the mean fitness value of BWO-IG achieved the minimum fitness value (shown in bold) as compared with most of the other approaches except for BIMFOHHO. The BWO algorithm performed better than BHHO in eight datasets. On the other hand, in comparison with BSC, it is clear that BWO-IG performed better in all datasets except for the CNS dataset. However, the results of BSC and BWO-IG in the case of the CNS dataset were very close.

In respect of the number of selected genes, BWO-IG achieved the best reduction rate across the nine datasets, as shown in Table 7 and Fig. 6**.**

**Table 7:** Comparison of average number of genes selected by proposed approaches and the other approaches.

Dataset	BWO	BIMFOHHO	BMFO	BHHO	BCS	BBA
**MLL**	5332.366	8751.698	8757.700	8762.981	8765.652	8777.732
**Colon**	917.6000	1241.267	1250.800	1254.880	1249.320	1267.224
**Leukemia**	2853.266	4495.058	4498.233	4502.112	4500.112	4511.878
**Leukemia_c3**	2890.100	4849.354	4847.267	4854.859	4850.447	4877.250
**Leukemia_c4**	2883.733	4810.852	4802.033	4814.290	4814.556	4843.589
**CNS**	2867.180	4647.421	4654.200	4662.254	4662.287	4673.589
**Ovarian**	6468.900	9744.542	9765.800	9772.899	9773.999	9776.882
**SRBCT**	761.2000	1636.579	1657.233	1666.274	1666.589	1668.100
**lymphoma**	1429.566	2509.009	2519.067	2529.148	2529.451	2539.777
Average	**2933.767**	5822.000	5829.013	5836.555	5835.128	5849.837

In the experiments, the effectiveness of the proposed approach in achieving a satisfactory balance between exploration and exploitation was demonstrated in a variety of ways. First, the BWO algorithm outperformed the other compared algorithms in terms of classification accuracy and selection size over most of the datasets. Furthermore, the proposed method was able to reduce the gap between maximum and minimum accuracy in an efficient manner, which allowed for quicker convergence. It is widely known that a consistent and fast rate of convergence leads to improved results, so this result is particularly promising. The convergence speed behavior curves were obtained by applying them to each dataset over 100 iterations in order to further measure the efficiency of the proposed process. The results for convergence speed showed that the BWO had the ability to change the exploitation search, which is comprised of two criteria: the number of iterations needed to achieve the optimal solution and the solution's initial starting point.

The proposed method was able to accelerate the rate of convergence during its search for the right solutions due to the improved balance. The most suitable features were chosen to ensure that the solutions found converged as far as possible. Thus, randomization-assisted exploration enabled the search of the solution space from a local perspective while also increasing the range of solutions.

## 8 Conclusion and future work

The key goal of this study was to propose a new approach for finding effective solutions and optimizing the GS process. In order to achieve this goal, the black widow optimization algorithm was applied as a potential solution to the GS problem. To propose a more effective approach for addressing GS problems. BWO is used as an optimizer in two ways: it was applied in its original form as well as hybridized with the iterated greedy algorithm (BWO-IG) to enhance its local search capabilities. Nine benchmark datasets from the gene expression data warehouse were used in the experiments. The results showed that the BWO-IG enhances the BWO's search capabilities; it can pick suitable genes and produce more reliable results than the BWO algorithm, whether in the accuracy rate or in the number of reduced genes. The performance of the proposed BWO-IG method was compared with that of five recent wrapper FS methods, namely, BIMFOHHO, BMFO, BHHO, BCS, and BBA. The comparison revealed that BWO-IG was significantly superior in terms of reducing the number of genes selected and getting highly accurate classification accuracy.

A thorough examination of dataset properties, such as dimensionality and dataset size, can be a future research path. Although the majority of feature selection (FS) research so far has focused on problems with dimensionality limited to a few thousand features, it is unclear how FS techniques will perform when faced with datasets comprising millions of features. Future research in the area will need to have a key focus on how to solve this scaling problem.

Additionally, expanding the application domain of suggested FS approaches beyond their original domain can produce insightful results. It is important to assess these techniques' effectiveness and applicability in various applications, including electroencephalogram (EEG) analysis, face recognition, and hyperspectral image processing. This broadening of the algorithm's scope can reveal fresh possibilities for enhancing various AI-driven applications.

Furthermore, improving FS algorithms to function in a multi-objective framework represents an interesting direction for future research. It may be possible to develop more flexible and adaptable feature selection strategies that are suited to the complexities of real-world AI challenges by researching algorithms that may simultaneously optimize many objectives, such as accuracy, interpretability, and robustness.

## Author contribution statement

Mohammed Alweshah: Conceived and designed the experiments; Performed the experiments; Analyzed and interpreted the data; Contributed reagents, materials, analysis tools or data; Wrote the paper. Yasmeen Aldabbas: Bilal Abu-Salih: Saleh Oqeil: Hazem Hasan: Saleh Alkhalaileh: Sofian Kassaymeh: Contributed reagents, materials, analysis tools or data; Wrote the paper.

## Data availability statement

Data will be made available on request.

## Declaration of competing interest

The authors declare that they have no known competing financial interests or personal relationships that could have appeared to influence the work reported in this paper.
